# Cryptococcal meningitis associated with increased adenosine deaminase in the cerebrospinal fluid

**DOI:** 10.1186/s40064-016-3767-z

**Published:** 2016-12-12

**Authors:** Yuji Tanaka, Kazuo Satomi

**Affiliations:** Department of Neurology, Gifu Municipal Hospital, 7-1 Kashima-Cho, Gifu City, 500-8513 Japan

**Keywords:** Cryptococcal meningitis, Adenosine deaminase, Cerebrospinal fluid, Follow up, Differential diagnosis

## Abstract

**Introduction:**

Clinically, increased cerebrospinal fluid (CSF) adenosine deaminase (ADA) level is an important diagnostic clue of tuberculous meningitis. However, increased CSF ADA level can be caused by other neurological diseases.

**Case description:**

We report a case of a 67-year-old woman with cryptococcal meningitis presented with increased ADA level of the CSF. In parallel with her recovery, the ADA level of CSF decreased steadily. This is the first case described the chronological change in CSF ADA level of the patient with cryptococcal meningitis in detail.

**Discussion and Evaluation:**

Clinically, increased CSF ADA level is an important diagnostic clue of tuberculous meningitis. However, previously, it was reported that increased CSF ADA level can be caused by other neurological diseases. In this case, the patient was diagnosed with cryptococcal meningitis, and the possibility of coinfection with tuberculous meningitis has been discarded by the negative PCR, negative cultures and the clinical course. In addition, the chronological change in CSF ADA level was useful for follow-up assessment.

**Conclusions:**

Cryptococcal meningitis should be considered for the differential diagnosis for diseases presented increased CSF ADA.

## Introduction

Clinically, increased cerebrospinal fluid (CSF) adenosine deaminase (ADA) level is an important diagnostic clue of tuberculous meningitis (TBM). However, increased CSF ADA level can be caused by other neurological diseases. We report of an older patient with cryptococcal meningitis associated with increased CSF ADA level, in which information on the chronological change in ADA level was useful for follow-up assessment.

## Case description

**Fig. 1:**
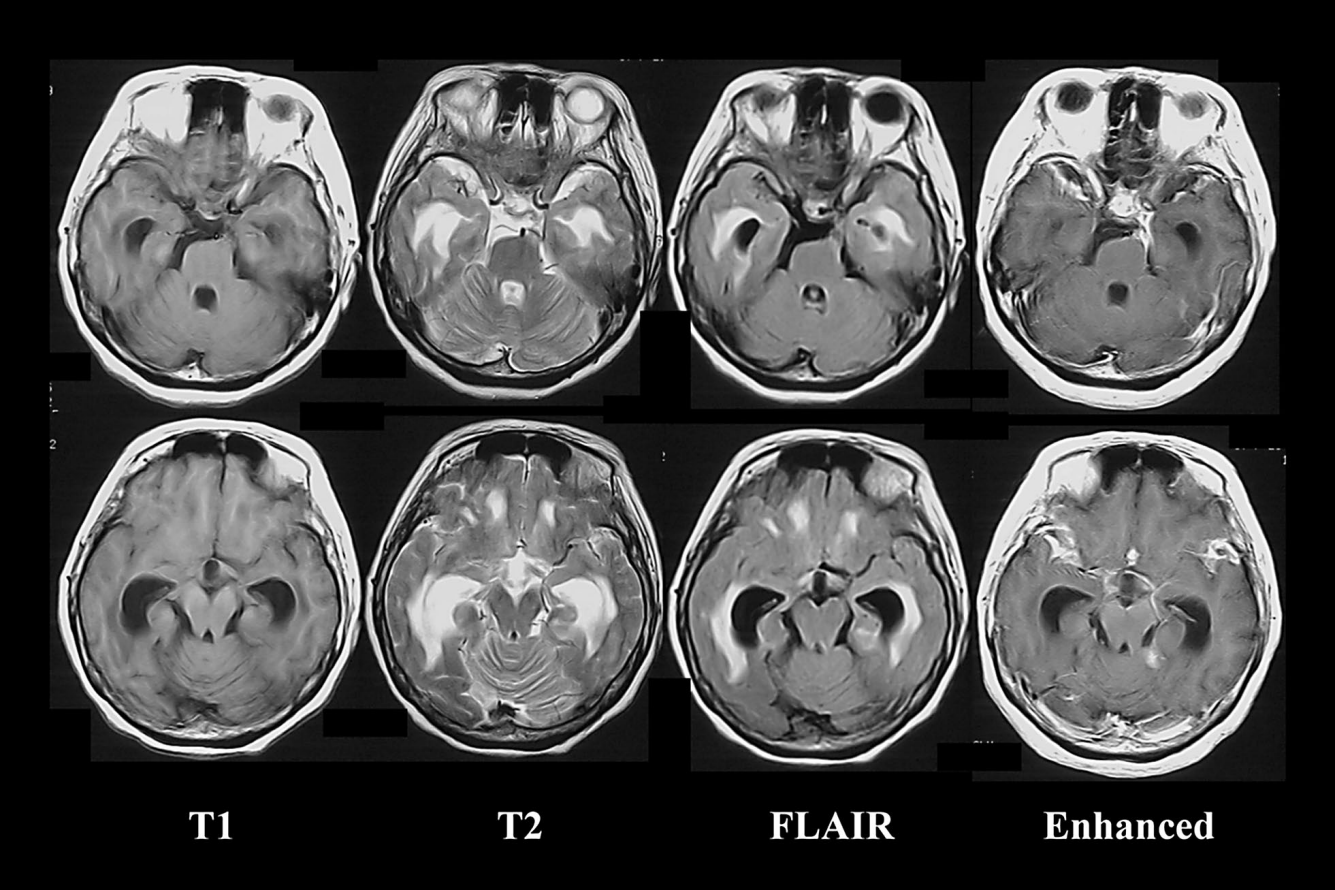
Brain magnetic resonance imaging on admission. The scan revealed hydrocephalus and an abnormal enhancement effect at the brain base

**Fig. 2:**
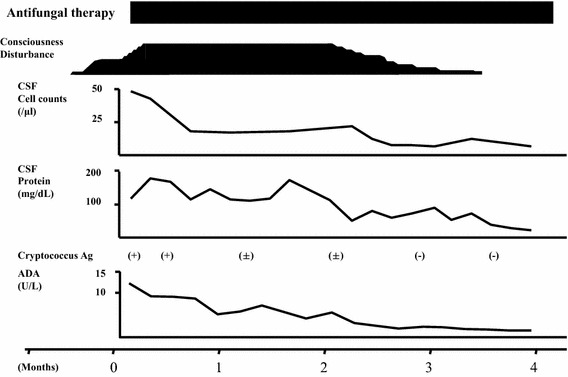
Clinical course. *ADA* adenosine deaminase, *CSF* cerebrospinal fluid

A 67-year-old woman was diagnosed with hypertension at age 30 years. Her family had no history of active tuberculosis, but the patient had bred a hill myna. Three months before admission to the hospital, the patient initially developed a slight fever and headache. After 3 months, the patient could not walk independently and her level of consciousness declined, for which the patient was admitted to our hospital. A neurological examination revealed reduced consciousness (Glasgow coma scale; E3 V3 M5) and meningeal irritation, hyperreflexia of all four-extremities, and positive Babinski response. Blood examination revealed normal findings, including inflammatory reactions, and negative results for human immunodeficiency virus antibody. In the CSF examination, the cell count was 46/µL (all lymphocytes); the protein level, 142 mg/dL (reference range 10–40 mg/dL); glucose level, 31 mg/dL (reference range 50–75 mg/dL); and ADA level, increased to 12.7 U/L (reference range <9.0 U/L) (Ribera et al. 1987). The India ink smear yielded a positive result, and Cryptococcus antigen was detected. A mycological culture of CSF revealed *Cryptococcus neoformans*. The mycobacterium cultures and polymerase chain reactions (PCR) of CSF, sputum, urine, and gastric juice repeatedly yielded negative results. Brain magnetic resonance imaging revealed hydrocephalus and an abnormal enhancement effect at the brain base (Fig. 1). Chest computed tomography revealed normal findings. According to these findings, cryptococcal meningitis was diagnosed. Administration of amphotericin B and flucytosine were initiated. At 1 month after admission, the CSF ADA level decreased to 5.6 U/L. At 4 months after admission, antimycotic therapy improved the patient’s symptoms and the Cryptococcal antigen in CSF disappeared. In parallel with her recovery, the CSF ADA level decreased steadily (Fig. 2).

## Discussion

We describe an older woman with increased CSF ADA level, who was diagnosed with cryptococcal meningitis rather than TBM. In addition, the chronological change in CSF ADA level was useful for follow-up assessment.

Clinically, increased CSF ADA level is an important diagnostic clue of TBM. Definite diagnosis of TBM requires detection of tubercle bacilli in CSF, and the most commonly used tests are CSF smears and/or cultures. Smear microscopy is quick and inexpensive but has low sensitivity (0–20%) (Tuon et al. 2010). Culture methods require up to 8 weeks and have variable sensitivity (10–90%) (Tuon et al. 2010). Moreover, CSF ADA level is a more sensitive indicator than PCR for the diagnosis of TBM (Rana et al. 2010). Additionally, CSF ADA level can be evaluated in resource-poor settings, including small general hospitals and non-specialized hospitals for infectious diseases, and contribute to a quick and accurate diagnosis.

ADA is an enzyme that catalyzes the deamination of adenosine, forming inosine in the process (Erel et al. 1998). The chief physiological function of ADA is related to lymphocytic proliferation and differentiation (Erel et al. 1998). Activity is found to be elevated in diseases with cell-mediated immune response and is a marker of cellular immunity (Erel et al. 1998). The mechanism of increased CSF ADA level has not been fully elucidated. One of the widely accepted theories is that increased ADA level is due to intrathecal synthesis after activation of monocyte–macrophages (Schutte et al. 2001). One reason is because ADA plays an important role in monocyte-to-macrophage maturation, and another is because the ADA (2), which is one of the two ADA isoenzymes, is the major contributor to increased CSF ADA level, probably reflecting the monocyte–macrophage origin of ADA (Schutte et al. 2001). These speculations suggest that increased CSF ADA level can be caused by TBM and other neurological diseases. However, in this case, the possibility of coinfection with TBM has been discarded by the negative PCR, negative cultures and the clinical course.

Previously, it was reported that increased CSF ADA level can be caused by other diseases such as sarcoid meningitis, meningeal involvement with leukemia or lymphoma, cerebral toxoplasmosis, cerebral infarction, neurosyphilis, listeria meningitis, and other aseptic meningitis (Nishida et al. 2007). In general, these are diseases that activate the lymphocytes to on turn activate the monocyte–macrophage system in a similar way to how granulomas are made. These include infectious and non-infectious causes. Thus, the differential diagnosis should include other neurological diseases.

Only two cases of cryptococcal meningitis associated with increased CSF ADA level have been described in the literature (Izumoto et al. 1991; Martínez et al. 1992). Because there was not the chronological change of CSF ADA level in previous reports, it was not clear whether increased CSF ADA level was caused by cryptococcal meningitis. In this case, the CSF ADA level at admission increased and in parallel with her recovery, the CSF ADA level decreased steadily. This is the first case described the chronological change in CSF ADA level of the patient with cryptococcal meningitis in detail. From the detailed observation of CSF ADA level, the determination of CSF ADA level was useful for follow-up assessment of cryptococcal meningitis.

## Conclusions

In conclusion, we report an older patient with cryptococcal meningitis associated with increased CSF ADA level, in which information on the chronological change in CSF ADA level was useful for follow-up assessment. Although the determination of CSF ADA level can be useful for early differential diagnosis of TBM, other neurological diseases such as cryptococcal meningitis should be considered.

## Abbreviations

ADA: adenosine deaminase
CSF: cerebrospinal fluid
TBM: tuberculous meningitis
PCR: polymerase chain reactions
